# Anomalie osseuse rare: brachydactylie non syndromique de type C

**DOI:** 10.11604/pamj.2016.25.220.11227

**Published:** 2016-12-06

**Authors:** Imad Ghozlani, Radouane Niamane

**Affiliations:** 1 Service de Rhumatologie, 1^er^ Centre Médico Chirurgical, Agadir, Maroc; 2 Service de Rhumatologie, Hôpital Militaire Avicenne, Maroc

**Keywords:** Brachydactylie, type C, non syndromique, Brachydactyly, type C, non-syndromic

## Image en médecine

La brachydactylie est un raccourcissement congénital des mains ou des pieds en rapport avec l’absence ou la petite taille des phalanges, des os métacarpiens ou métatarsiens. Il s’agit d’une anomalie peu fréquente, décelable aisément sur le plan clinique, mais pour laquelle un complément radiologique est indispensable. Nous présentons le cas d’un patient de 40 ans qui a consulté pour une douleur mécanique de la base du pouce droit en rapport avec une arthrose Trapézo-métacarpienne dite rhizarthrose et chez qui l’examen a trouvé fortuitement un raccourcissement des 2^ème^, 3^ème^ et 5^ème^ doigts des deux mains (A). Les pieds étaient épargnés. Il n’y avait pas d’autres atteintes dysmorphiques (oculaire, nasale, auriculaire ou digestive). L’interrogatoire a permis de révéler la même atteinte uniquement chez la mère sans notion de consanguinité. La radiographie standard de face des deux mains a montré une brachymésophalangie des deuxièmes, troisièmes et cinquièmes rayons (B). Ce type de brachydactylie est plus complexe. Le quatrième rayon est épargné, apparaissant ainsi comme le doigt le plus long. La brachydactylie peut être classée en cinq types différents de A à E. La radiographie standard est l’examen clé pour la caractérisation des brachydactylies, précisant la topographie et le type de l’atteinte. Plusieurs formes de brachydactylie sont causées par des mutations des gènes codant les composants de la Protéine Osseuse Morphogénétique. Les brachydactylies peuvent également s’intégrer dans un grand nombre de syndrome poly malformatifs. Notre patient présente une brachydactylie isolée non syndromique de type C se transmettant selon un mode autosomique dominant.

**Figure 1:**
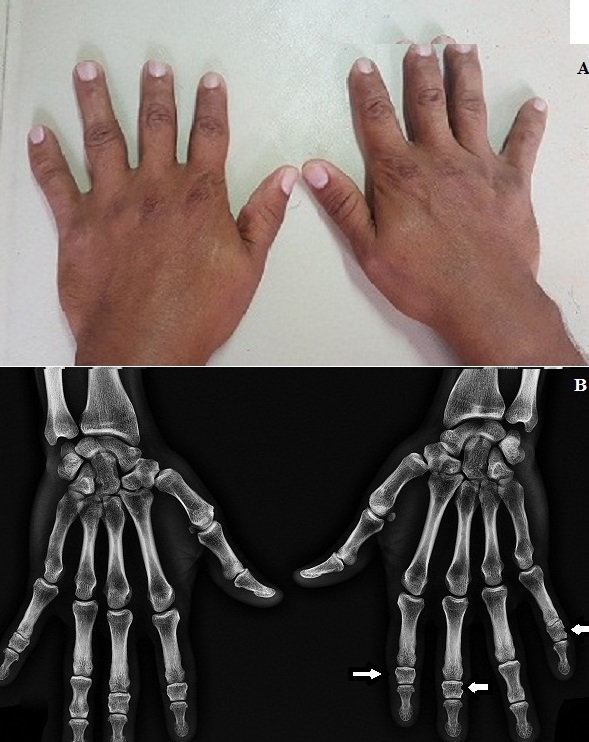
A) raccourcissement des 2^ème^, 3^ème^ et 5^ème^ doigts des deux mains; B) radiographie standard de face des deux mains montrant une brachymésophalangie des 2^ème^, 3^ème^ et 5^ème^ rayons

